# Auricular acupressure for adverse events following immunization related to COVID-19 vaccine injection: study protocol for a multicenter, three-arm, blinded randomized controlled trial

**DOI:** 10.1186/s13063-021-05837-x

**Published:** 2021-11-27

**Authors:** Qinwei Fu, Hui Xie, Li Zhou, Xinrong Li, Yang Liu, Min Liu, Chaoyu Wang, Xiaocen Wang, Zhiqiao Wang, Jinfan Tang, Huan Xiao, Zhiyong Xiao, Jing Zhou, Chengzhi Feng, Li Wang, Zhimin Ao, Xi Chen, Chang Su, Xuanyu Wu, Maolan Zhao, Sihan Hu, Hanwen Lin, Jiali Huang, Guo Xu, Qinxiu Zhang, Luyun Jiang

**Affiliations:** 1 Hospital of Chengdu university of Traditional Chinese Medicine, Chengdu university of Traditional Chinese Medicine, Chengdu, 610075 China; 2 Du Jiang Yan Medical Center, Du Jiang Yan, 611830 China; 3 Acupuncture and Tuina School, Chengdu University of Traditional Chinese Medicine, Chengdu, 610075 China; 4 Eye School of Chengdu University of Traditional Chinese Medicine, Chengdu, 610075 China; 5 School of Medical and Life Sciences, Chengdu University of Traditional Chinese Medicine, Chengdu, 611137 China

**Keywords:** Auricular acupressure, COVID-19, Vaccine, Adverse events, Randomized controlled trial, Protocol

## Abstract

**Background:**

Some pain, fatigue, and gastrointestinal adverse events were observed in potential association with injection of COVID-19 vaccines, while there was no preventive intervention for it. We aim to investigate the efficacy of auricular acupressure (AA) therapy in preventing and relieving AEFI after injection of COVID-19 vaccine.

**Methods:**

The study design is a randomized, multicentre, three-arm controlled, single-blind trial. Participants meeting the inclusion criteria will be advertised and enrolled and assigned in the medical institutions randomly for post-injection observation. No less than 360 participants will be randomized into one of three groups: auricular acupressure group, sham auricular acupressure group, and wait-list group. Interventions will be performed immediately and will happen 4 to 5 times per day for 5 days. The primary clinical outcomes will be quality and quantity evaluation among participants who reported any AEFI and who reported local pain at injection site. Secondary outcomes will concern headache, muscle and (or) joint pain, fatigue, nausea, vomiting, diarrhoea, and other potential events. All the outcomes will be assessed at baseline and 1, 3, 5, 7, and 14 days after the injection. Both intention-to-treat and per-protocol analyses will be performed, with significance level determined as 5%.

**Discussion:**

Results of this trial will help to clarify the value of auricular acupressure therapy in preventing and relieving overall and certain adverse events following immunization after injection of COVID-19 vaccine.

**Trial registration:**

China Clinical Trial Registry (ChiCTR) (ChiCTR2100043210). Registered on 8 February, 2021.

## Administrative information

**Table:** 

Title {1}	Auricular Acupressure for Adverse Events Following Immunization Related to COVID-19 Vaccine Injection: Study Protocol for a multicenter, three-arm, blinded randomized controlled trial
Trial registration {2a and 2b}.	This trial was registered in the China Clinical Trial Registry (ChiCTR) (ChiCTR2100043210) on 8th February, 2021.
Protocol version {3}	Date: 3^rd^ Feb, 2021. Version: V2.0
Funding {4}	1. Xinglin Scholars Scientific Research Promotion Plan of Chengdu University of Traditional Chinese Medicine-Innovation team of traditional Chinese medicine otorhinolaryngology discipline, natural science (No. XKTD2021003); 2. Scientific Project of Hospital of Chengdu University of Traditional Chinese Medicine (No. 21LL2X03); 3. Chengdu Medical Research Project (2021002); 4. National College Students’ Innovation and Entrepreneurship Project (202110633016); 5. Graduate Scientific Research Innovation Project of Clinical Medical College of Chengdu University of Traditional Chinese Medicine (2020ZS204).
Author details {5a}	a Hospital of Chengdu university of Traditional Chinese Medicine, Chengdu university of Traditional Chinese Medicine, Chengdu, China, Postal code: 610075. b Du Jiang Yan Medical Center, Du Jiang Yan, China, Postal code: 611830. c Acupuncture and Tuina School, Chengdu University of Traditional Chinese Medicine, Chengdu, China, Postal code: 610075. d Eye School of Chengdu University of Traditional Chinese Medicine, Chengdu, China, Postal code: 610075. e School of Medical and Life Sciences, Chengdu University of Traditional Chinese Medicine, Chengdu, China, Postal code: 611137.
Name and contact information for the trial sponsor {5b}	Qinxiu Zhang*: Hospital of Chengdu university of Traditional Chinese Medicine, Chengdu university of Traditional Chinese Medicine, Chengdu, China, Postal code: 610075 & School of Medical and Life Sciences, Chengdu University of Traditional Chinese Medicine, Chengdu, China, Postal code: 611137. zhqinxiu@163.com. Luyun Jiang*: Hospital of Chengdu university of Traditional Chinese Medicine, Chengdu university of Traditional Chinese Medicine, Chengdu, China, Postal code: 610075. jly666 @163.com.
Role of sponsor {5c}	Study sponsors are also corresponding authors in this study, and they are involved in study design, collection, management, analysis, interpretation of data, writing of the report, and the decision to submit the report for publication. They will have ultimate authority over any of these activities. However, the funders have no role in these activities.

## Introduction

### Background and rationale {6a}

As of October 5, 2021, a total of 234 million people were confirmed infected with SARS-CoV-2, including 4.8 million deaths worldwide [1]. Developing immunity through COVID-19 vaccination provides a reduced risk of being infected and helps to fight the virus if exposed, which can save uncounted lives and give us a pathway out of the global disaster [2, 3]. Researches showed that novel coronavirus-19 vaccines (NCV) were of considerable efficacy, and improvement on efficacy, especially long-term protection, and exploration of new species of vaccines are being conducted [4–8]. Some new variants of COVID-19 have been emerging, which may exacerbate COVID-19 symptoms, while researches reveal that many still with good immunogenicity [9, 10]. However, some studies show concern on prevention efficacy in the situation [11, 12]. For this, some researchers argue for strengthening injection of current NCV; simultaneously, new NCVs are being developed by countries [13, 14]. According to the WHO, till October 5, 2021, 124 of NCV products are in clinical development, and 194 are in pre-clinical development worldwide, with the top three types of NCVs listed as protein subunit (43, 35%), viral vector (non-replicating, 18, 15%), and DNA (12, 10%) [15].

Clinical experiments observed some adverse events following immunization (AEFI) in potential association with injection of NCVs, though most of them disappeared after several or a dozen days without special medical intervention. Among them, most frequent events were localized reactions (such as pain, sore, swelling, and itching), followed by fatigue, fever (some with chills), headache, myalgia, dizziness, and nausea (some with vomiting) [4–6, 16–19]. Mechanically, most AEFI are induced by hypersensitivity of organs or immune response to vaccine, which is more obvious in strengthening injection of NCV, especially [20, 21].

Till now, no study has been made on preventive intervention for AEFI after injection of NCVs or on other vaccines. Considering the huge medical burden and potential hesitancy, anxiety, or even rejection of some people toward NCVs injection, an appropriate preventive intervention is needed, which can provide better comfort and higher quality of lives in short terms both physiologically and psychologically [17–19, 22]. We published structured protocol summary of this study for brief introduction, with full Chinese protocol attached [23]. For months, some patients, researchers and people who were interested mailed us about more details of the protocol and study in English worldwide. As a result, we conduct a full-length protocol of the study.

Auricular acupressure (AA) therapy is non-invasive and non-pharmaceutical, easily taught and self-implemented, inexpensive and with nearly no side effect. By pressing at auricular acupoints, the therapy can stimulate the meridians, influence the release of neurotransmitters that transmit signals along neurons, and regulate the function of endocrine and viscera [24, 25]. Researches showed that AA has been successfully used for surgery pain, hypertension, insomnia, anxiety, depression, relieving nausea, vomiting, constipation, fatigue, and lack of appetite related to chemotherapy [24–29]. Weighing up the harm-benefit balance, evidence suggests potential benefits of AA therapy on preventing and relieving NCV-related AEFI.

This study was designed to investigate dynamically whether AA therapy was effective in preventing and (or) relieving overall and certain AEFI of NCV after injection compared with sham auricular acupressure (SAA) and wait-list (WL) control.

## Objectives {7}

To test whether AA therapy was effective in preventing and (or) relieving AEFI of the NCV after injection compared with SAA and WL control.

## Trial design {8}

This study will be an explorative, multicentre, three-arm, single-blind, prospective randomized controlled trial. Participants will be randomized in 1:1:1 ratio to AA group, SAA group, and WL group.

## Methods: participants, interventions, and outcomes

### Study setting {9}

**Fig. 1:**
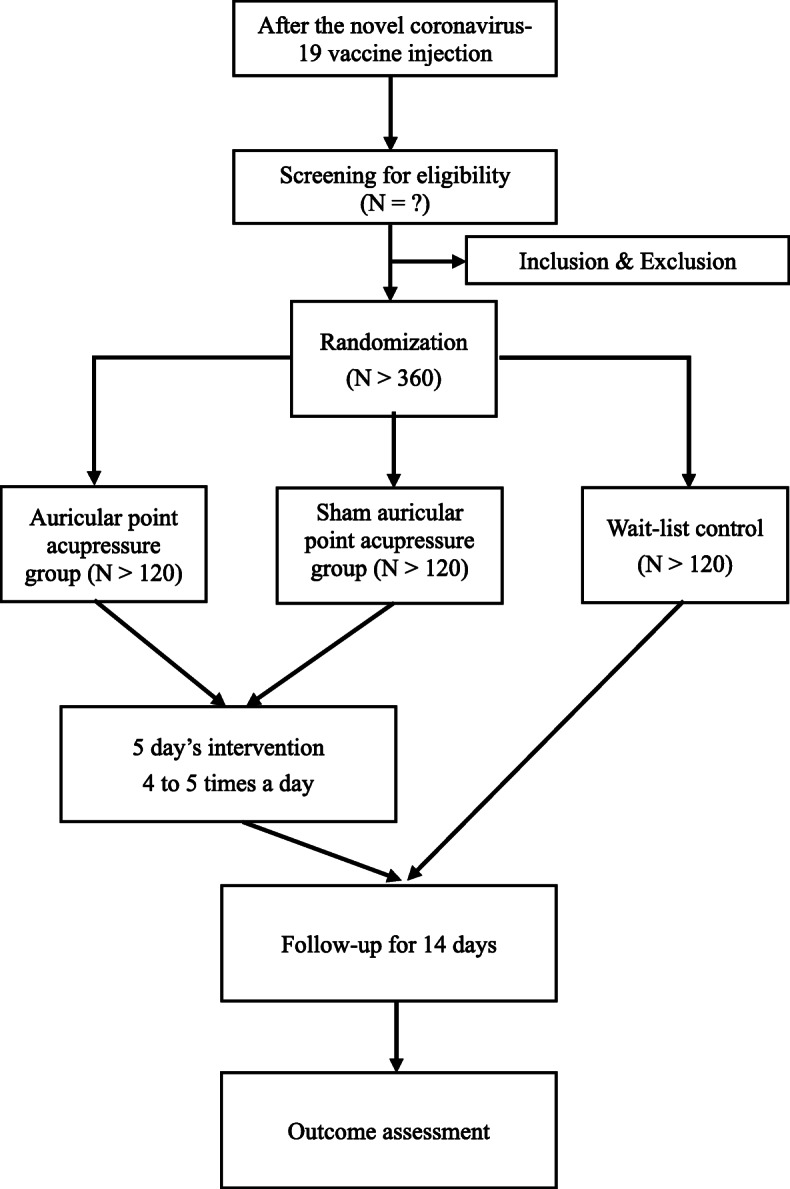
The flow chart of the trial

This study is approved by the Ethics Committee of Hospital of Chengdu University of Traditional Chinese Medicine (2021KL-015), and jointly funded. No less than 360 participants for one type of NCV will be advertised and enrolled and randomly assigned to one of the three groups. Participants will be advertised and enrolled in four centres in Chengdu, China, including Hospital of Chengdu university of Traditional Chinese Medicine, Caotang Community Health Service Center, Xi’an Road Community Health Service Center, and Wenjiang School Hospital of Chengdu University of Traditional Chinese Medicine. To examine the effectiveness of AA therapy in preventing and (or) relieving AEFI related to NCV, measurements will be taken once the injection is completed, and the participants will be followed after 1, 3, 5, 7, and 14 days after NCV injection. Participants will be asked to accept assessments at baseline. This randomized controlled clinical trial began recruitment on March 17, 2021, and the anticipated completion date is February 2022. The primary outcome are quality and quantity evaluation among participants who reported any AEFI and who reported local pain at injection site. The flow chart of the trial is shown in Fig. 1. Informed consent is obtained from each participant. The protocol is reported following the *Trials* structured template [30].

### Eligibility criteria {10}

#### Inclusion criteria

Vaccinators will be screened strictly for meeting the criteria of the NCV injection shortly before the injection (during registration) [31]. Patients will be included if they meet all the following criteria:

1. Conforming to the conditions of the injection without contraindication, and completing the NCV injection within 24 h before screening
2. No headache, muscle or joint pain, fatigue, diarrhoea, nausea, or vomiting experienced (no more than 4 scores, VAS) within 3 days before the injection, and no diseases (diagnosed) presenting the symptoms above
3. No redness, swelling, injury, or infection on the skin or soft tissue of bilateral ears
4. No history of alcohol and adhesive tape contact allergy
5. Able to complete the follow-up questionnaires independently online or by phone
6. Agree to participate and sign the informed consent, and abide by precautions after the injection and requirements of AA therapy

In addition, it should be pointed out that due to the emergency of NCV injection and the good safety of AA therapy, we do not restrict ages and genders among participants. We will conduct sub-groups for different ages for comparison.

#### Exclusion criteria

Patients will be excluded if they have one or more of the following:

1. Not suitable to be vaccinated because of contraindication or were in cautious condition
2. Have participated in other trials within 4 weeks before the start of this study
3. With headache, muscle or joint pain, fatigue, diarrhoea, nausea, or vomiting experienced (serious than 4 scores, VAS) within 3 days before the injection, or with diseases (diagnosed) presenting the symptoms above
4. Pregnant or lactating women
5. With other serious primary diseases and psychosis

#### Withdraw from the trial

Participants will be allowed or asked to drop out from the trial if they:

1. Are lost to follow-up
2. Become pregnant
3. Develop serious adverse event (SAE)

Participants can withdraw from this clinical trial at any time. The date and reason for withdrawal should be stated. If possible, all subjects withdrawing from the study should continue to be followed up regularly on a measurement schedule with a final assessment for intention-to-treat analysis.

### Who will take informed consent? {26a}

Paper informed consent will be obtained from eligible patients by pre-appointed researchers (C. W., Z. X., H. L. and C. S.) during enrolment but before randomization.

### Additional consent provisions for collection and use of participant data and biological specimens {26b}

No biological sample will be collected in our study.

### Interventions

#### Explanation for the choice of comparators {6b}

Auricular acupoints for AA group and SAA group were selected according to relevant international and national standards of China, expert consultation, our experience, and based on potential AEFI of the NCVs from previous studies [4–6, 16–19, 32–34].

#### Intervention description {11a}

**Fig. 2:**
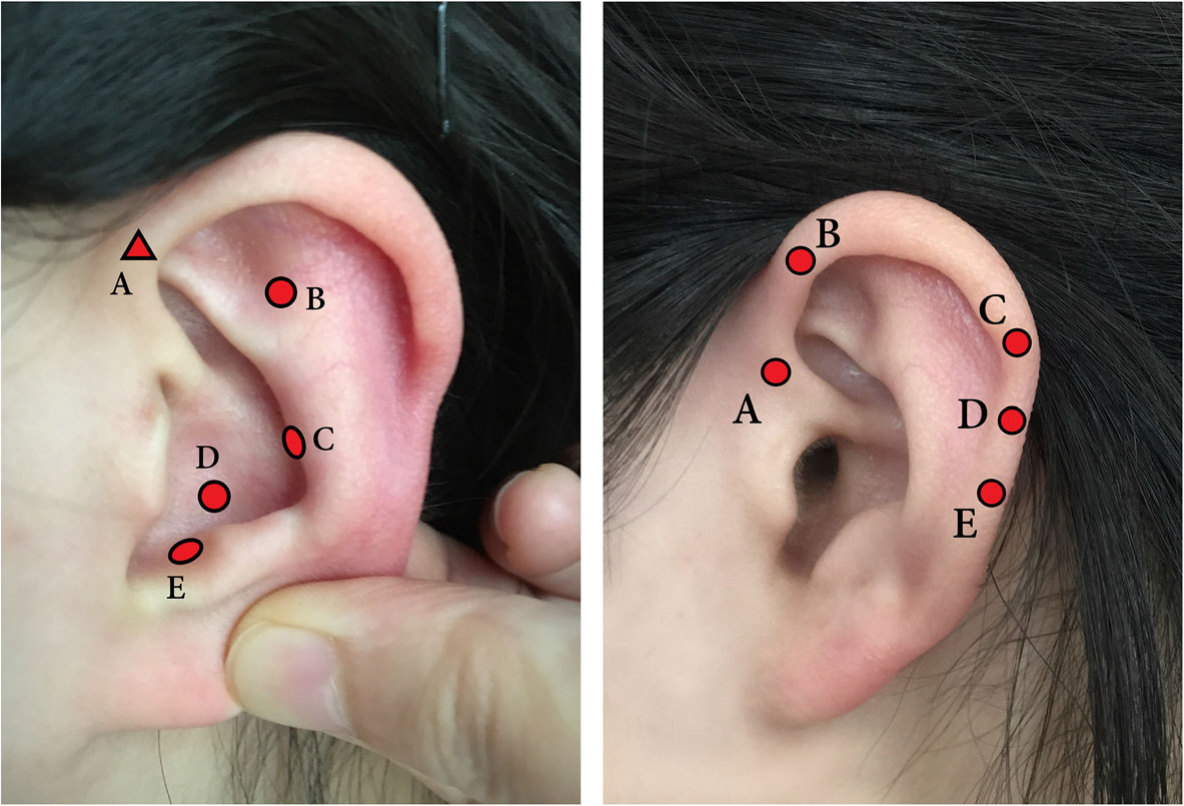
Auricular acupoints applied for AA and SAA groups. Circular marks indicate the acupoints are located at the outer surface, and triangle mark indicates the acupoint is at inside surface. The left picture is the distribution of auricular acupoints applied for AA group, including **A** [Jiaogan (sympathetic, AH6a)], **B** [Shenmen (TF4)], **C** [Pi (Spleen, CO13)], **D** [Xin (Heart, CO15)], and **E** [Pizhixia (Subcortex, AT4)]. The picture in the right is the distribution of auricular acupoints applied for SAA group, including A [Niaodao (Urethra, HX3)], B [Gangmen (Anus, HX5)], C [Helix 1 (HX9)], D [Helix 2 (HX10)], and E [Helix 3 (HX11)].

All researchers received enough pre-experiment training, and acupuncturists who performed AA and SAA were trained for at least 4 years with clinical experience. Five auricular acupoints were applied for both AA and SAA groups, bilaterally, including Shenmen (TF4), Pi (Spleen, CO13), Xin (Heart, CO15), Pizhixia (Subcortex, AT4), and Jiaogan (sympathetic, AH6a) for AA group, and Gangmen (Anus, HX5), Niaodao (Urethra, HX3), Helix 1 (HX9), Helix 2 (HX10), and Helix 3 (HX11) for SAA group (Fig. 2 and Table 1).

**Table 1:** Auricular acupoints selected in our protocol (with location and function)

Group	Auricular acupoints	Anatomical location	Main function(s) according to TCM
For the auricular acupressure group	Shenmen (TF4)	The upper part of the posterior 1/3 of the triangular fossa of auricle (a Area 4 of auricle triangular fossa)	Insomnia, abnormal sweating, pain, cough, asthma, vertigo, hypertension, allergy, withdrawal syndrome, epilepsy
	Pi (Spleen, CO13)	Below the BD line of the auricle and above the posterior part of the concha cavity (Concha 13 area of auricle)	Diarrhoea, abdominal distention, constipation, loss of appetite, functional uterine bleeding, excessive leucorrhea, inner ear vertigo, edema, visceral ptosis
	Xin (Heart, CO15)	In the middle of the concha cavity of the auricle (concha 15 area of auricle)	Tachycardia, arrhythmia, angina pectoris, spontaneous sweating, night sweats, hysteria, sore tongue, palpitation, insomnia, forgetfulness
	Pizhixia (Subcortex, AT4)	On the inner side of the opposite tragus of the auricle (zone 4 of the opposite tragus of the auricle)	Pain, neurasthenia, gastric ulcer, pseudomyopia, diarrhoea, hypertension, coronary heart disease, arrhythmia, insomnia
	Jiaogan (sympathetic, AH6a)	At the junction of the anterior segment of the lower foot of the opposite ear wheel of the auricle and the inner edge of the ear wheel (anterior segment of zone 6 of the opposite ear wheel)	Autonomic nervous function diseases, visceral pain
For the sham auricular acupressure group	Gangmen (Anus, HX5)	In front of the triangular fossa of the auricle (ear wheel area 5 of the auricle)	Haemorrhoids, anal fissure
	Niaodao (Urethra, HX3)	At the anterior and upper part of the protrusion of the foot of the auricle (ear wheel area 3 of the auricle)	Frequent urination, urgent urination, urinary pain, urinary retention
	Helix 1 (HX9)	Below the tubercle of the auricle (ear wheel area 9 of the auricle)	Tonsillitis, upper respiratory tract infection, fever
	Helix 2 (HX10)	Just below the Helix 1/ HX9 (ear wheel area 10 of the auricle)	
	Helix 3 (HX11)	Just below the Helix 2/ HX10 (ear wheel area 11 of the auricle)	

**Fig. 3:**
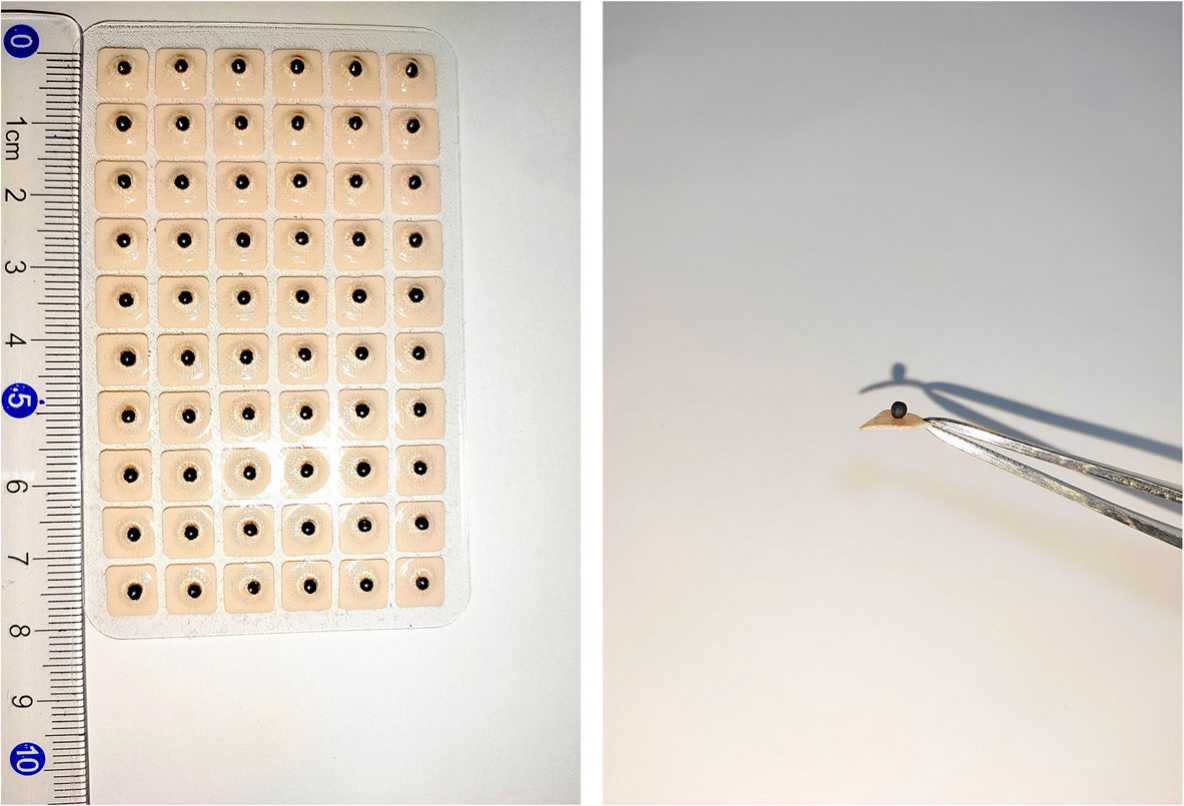
The Semen Vaccariae tapes in our study

Tools (model “Ziyu”; He’s Medical Device Co., Ltd, Henshui, China) applied for AA and SAA were Semen Vaccariae (about 2 mm in diameter), which were black, round, hard, and very small without special smell, covered with small tapes (about 0.8 × 0.8 mm2) (Fig. 3).

Surface of the auricular acupoints, marked on stick figures with two protocols associated with the sequence numbers for AA and SAA, was cleaned and dried with disinfection swabs, and Semen Vaccariae tapes were pasted in the acupoints by the acupuncturists and pressed with fingers to achieve sensation of soreness or distention.

In addition, the participants in AA and SAA groups were informed to press the tapes by themselves for 1 min vertically and appropriately to achieve the sensation, with a duration of 4 to 5 times a day and 5 days in total. The content above was written in Attention After AA Therapy, which were distributed to the participants with the individual sequence number recorded.

#### Criteria for discontinuing or modifying allocated interventions {11b}

Participants will be allowed or asked to discontinue the trial if they meet any item of the withdraw criteria. In addition, due to the pandemic, they can discontinue the trial at any time with the date and reason recorded.

#### Strategies to improve adherence to interventions {11c}

All of the AA and SAA interventions and service will be free for the participants, and they were informed at the beginning of the trial that two sessions of AA therapy, based on individual health condition, would be provided for free upon accomplishment of the study.

#### Relevant concomitant care permitted or prohibited during the trial {11d}

Implementing AA or SAA therapies for the AEFI will not require alteration to usual care pathways (including use of any medication) and these will continue for both trial arms.

#### Provisions for post-trial care {30}

Not applicable because AA therapy is non-invasive and non-pharmaceutical, easily taught and self-implemented, inexpensive, and with nearly no side effect.

### Outcomes {12}

All outcomes will be accessed and evaluated from the participants online or by phone in 1, 3, 5, 7, and 14 days after NCV injection, which they thought might be related to the injection. Primary outcomes are percentages of participants who reported any AEFI and who reported local pain at injection site, with their severity evaluated by visual analogue scale (VAS) scores of 0–10. Secondary outcomes include percentages of participants who reported headache, muscle and (or) joint pain, fatigue, nausea, vomiting, and diarrhoea, together with other AEFI reported by participants but not listed above, and with their severity evaluated by the VAS scores, similarly.

### Participant timeline {13}

The participant timeline is listed in Table 2.

**Table 2:**
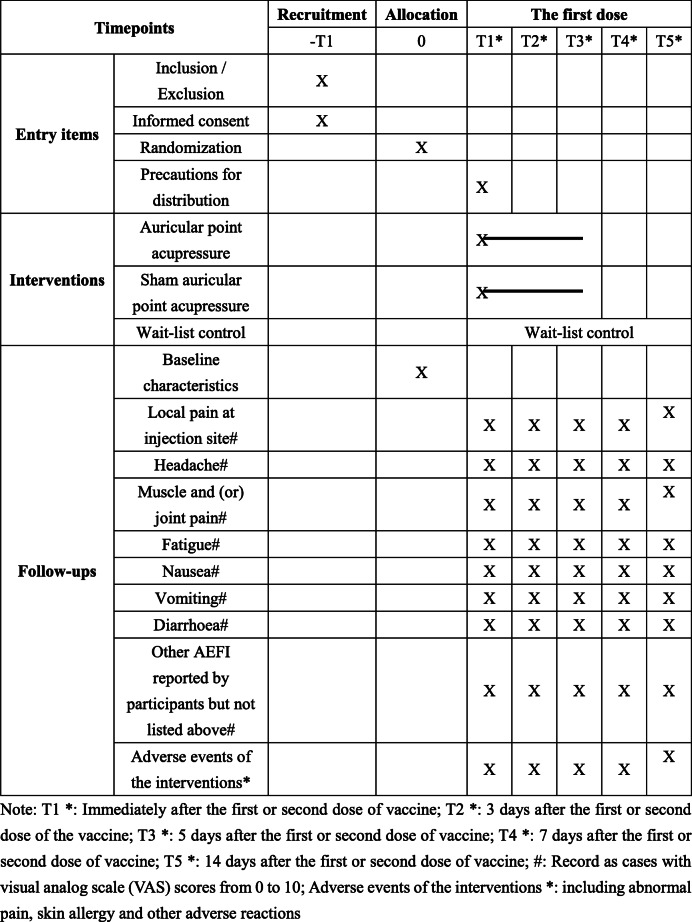
The participant timeline of our study

Note: T1*, immediately after the first or second dose of NCV; T2*, 3 days after the first or second dose of the NCV; T3*, 5 days after the first or second dose of NCV; T4*, 7 days after the first or second dose of NCV; T5*, 14 days after the first or second dose of NCV; #, record as cases with visual analogue scale (VAS) scores from 0 to 10; Adverse events of the interventions*, including abnormal pain, skin allergy, and other adverse reactions

### Sample size {14}

No less than 360 participants will be randomized in 1:1:1 ratio to each group. This is a protocol for an explorative study; therefore, a formal sample size is unavailable and not calculated.

### Recruitment {15}

We will recruit participants through leaflets and posters when waiting for the injection, and will be advertised, recruited during remaining in the medical institutions for post-injection observation (at less 30 min). Trial investigators will examine respondents in the observation areas for eligibility screening.

## Assignment of interventions: allocation

### Sequence generation {16a}

After confirmation for eligibility and collection of written informed consents, participants will be randomized through a random allocation software (The Hefei Big Orange Software Technology Co., Ltd, V 2.1.8), and be allocated to the AA, SAA, and WL groups in a 1:1:1 ratio with stratifying by centres.

### Concealment mechanism {16b}

Sequentially numbered sequences with combination of numbers and letters will be performed and recorded by registrars to conceal the random sequence for the participants, outcome assessors, and statisticians.

### Implementation {16c}

The sequences will be generated and concealed by trained research assistants.

## Assignment of interventions: blinding

### Who will be blinded {17a}

The participants in the AA group and SAA group, outcome assessors, and statisticians will be blinded, while participants in the WL group and the acupuncturists will not.

### Procedure for unblinding if needed {17b}

As for procedure for unblinding, the research assistants will record, unblind, and state it through the registration system if it is needed.

## Data collection and management

### Plans for assessment and collection of outcomes {18a}

In addition to the primary and secondary outcomes in our protocol, AEFI with extra specific medical intervention will be recorded. Adverse events associate with the AA therapy for AA and SAA groups will also be recorded, with cases, severity, measurement, and outcomes. Follow-ups will be performed by trained and blinded evaluators at baseline and 1, 3, 5, 7, and 14 days after the injection. However, due to the large number of vaccinations in facilities, especially in the waiting and observation areas, time-consuming questionnaires and indicators with more complexity will not be implemented. Data will be collected by a specialized research assistant, who will be responsible for the patient consents and case report forms.

### Plans to promote participant retention and complete follow-up {18b}

Participants who discontinue or deviate from the intervention protocols will be connected online or by phone, with their reasons for discontinue or deviate recorded. They will still be encouraged to report the AEFI in themselves during follow-ups and be told that two sessions of free AA therapy, based on individual health condition, would be provided for free upon accomplishment of the study

### Data management {19}

Data will be entered electronically in our study with double entry and range checks by trained and blinded researchers and will be recorded with good security. Soft copies of the research materials will be saved on a storage device, and all data will be maintained in a locked storage and only those with permission from the principal investigators will be able to access the data. All the retained data will be coded to identify the patients instead of personal information. The principal investigators will be given access to the cleaned datasets. In order to ensure confidentiality, the project team members will be blinded to any identifying participant information.

### Confidentiality {27}

During and after the trial, personal information about potential and enrolled participants will be kept in a pre-designated researcher of our study with good confidentiality. Data collected during the course of the research will be kept strictly confidential and only accessed by members of the trial team. Participants will be allocated an individual trial identification number when their details are stored on a secure database.

### Plans for collection, laboratory evaluation, and storage of biological specimens for genetic or molecular analysis in this trial/future use {33}

No biological sample will be collected in our study.

## Statistical methods

### Statistical methods for primary and secondary outcomes {20a}

Descriptive analyses will be made of frequencies and percentages for the qualitative variables, and overall *p* values will be estimated by *χ*^2^ or fisher *χ*^2^ test as appropriate with the Bonferroni approach. Pairwise comparisons will be performed as differences of percentages (DP), with 95% CIs of the differences calculated by R (V4.0.1). Estimations for *p* values of the pairwise comparisons will be performed by *χ*^2^, continuous or Fisher *χ*^2^ test as appropriate in SPSS (V23.0). Two-tailed tests with *p* < 0.05 define statistically significant for all analyses.

### Interim analyses {21b}

Not applicable in our protocol. Interim analysis and stopping guideline were not planned in our study because intervention and follow-up time is not long.

### Methods for additional analyses (e.g. subgroup analyses) {20b}

In additional analyses, the dosage of NCVs, genders, ages, and any experience of receiving AA therapy will be determined for each treatment arm.

### Methods in analysis to handle protocol non-adherence and any statistical methods to handle missing data {20c}

Clinical outcomes will be analysed in both intention-to-treat (ITT) and per-protocol (PP) participants, and missing values will be imputed by the last-observation-carried forward method. Results of participants who received AA (auricular acupressure), SAA (sham auricular acupressure) or WL (wait-list) observation, and with baseline details recorded will be processed with ITT analyses, while those who complete all the 14-day evaluation and with the interventions applied correctly will be analysed with PP sets.

### Plans to give access to the full protocol, participant-level data, and statistical code {31c}

Results of this study will be disseminated through publication (with no restriction) and academic conferences. The datasets used and analysed during the current study are available from the corresponding author on reasonable request.

## Oversight and monitoring

### Composition of the coordinating centre and trial steering committee {5d}

Coordinating centre, steering committee, endpoint adjudication committee, and data management team were gathered for our study. Coordinating centre and steering committee are responsible for general monitoring and providing organizational support. They also involved in study design, interpretation of data, writing of the report, and the decision to submit the report for publication. Endpoint adjudication committee and data management team are responsible for providing day to day support for the trial, including participant evaluation, collection, management, withdraws, analysis, and follow-up.

### Composition of the data monitoring committee, its role, and reporting structure {21a}

Data monitoring committee is served by the moderator of different participating units or a doctor from the same unit who is familiar with the relevant clinical field, has a graduate degree, has participated in RCT research or has participated in GCP-related training, and has the corresponding knowledge. Inspectors are required to undergo the necessary training to understand their duties before the project begins. Data monitoring committee should follow standard operating procedures and supervise clinical studies to ensure that they are carried out according to protocol. The committee is independent from the sponsor and with no competing interests.

### Adverse event reporting and harms {22}

Evidence suggest that SAEs are not anticipated in the AA and SAA therapy, and potential but very limited and minor adverse events may be local allergic reacts on the ear skin due to medical tape of AA and SAA therapy (itching, redness, and pain). And that they will be recorded and reported to the coordinating centres, principle investigators, and relevant regulatory bodies as required indicating expectedness, serious-ness, severity, and causality.

### Frequency and plans for auditing trial conduct {23}

To ensure that the quality of the data are in accordance with the protocol, the regular day-to-day monitoring during follow-ups will be carried out by clinical research investigators, who will be blinded to the allocation. The investigators will examine whether the recruitment procedures and data recording followed the protocol in the case report forms, and discuss the unpredictable changes in the research process, such as changes to the eligibility criteria, treatment regimens, or duration of follow-up with independent researchers and statisticians. In the case of SAEs or crucial issues, the investigator will determine whether the events are acceptable or whether it is necessary to change or terminate the trial.

### Plans for communicating important protocol amendments to relevant parties (e.g. trial participants, ethical committees) {25}

Investigators, trial participants, ethical committee, trial registries, journals, and regulators will be informed about important protocol modifications by e-mail or by phone if happened. Any deviation from the protocol will be fully documented using a breach report form.

## Discussion

The study is a multicentre, single-blinded, three-arm, randomized clinical trial to evaluate the efficacy and safety of AA therapy in preventing and (or) relieving overall and certain AEFI of NCV compared with SAA and WL control. Some AEFI were observed in potential association with injection of NCV, while there is no preventive intervention for it. As a result, it would be the first of such research.

In traditional Chinese medicine, all the twelve meridians pass through the ear, and AA therapy promotes the circulation of Qi and blood and regulates the balance of Yin and Yang and the functions of the viscera [32]. If the outcome from this study favours AA therapy with good preventing and (or) relieving effects in AEFI of NCV, larger scale of studies may be proposed. Furtherly, AA therapy may also have the potential to be applied for preventing or relieving AEFI of other types of vaccine, and more researches are needed. In addition, AA therapy is self-implementable and is also beneficial for relieving anxiety associated with the AEFI, especially during the pandemic.

Main limitation of this study is that there are some risks that small number of participants may know certain effects of different auricular points when communicating with others, or when visiting a doctor. These may decrease the reliability of blinding methods and credible level of the study. In addition, blinding for the acupuncturists may not applicable due to their expertise. For this, we will record it if a participant has revived AA therapy for other discomforts, and encourage participants do not to communicate with others about the vaccariae tapes pasted on their auricular points.

In conclusion, this study is an explorative study and will firstly provide the basis for the effectiveness and safety of AA therapy on AEFI related to NCV injection and would explore the possibility of AA therapy in the management of AEFI related to injection of other vaccines.

## Trial status

Protocol version: V2.0. Date of the version: 3 Feb 2021. The participants are currently being recruited for the present study since March 17, 2021, and recruitment is anticipated to be completed in March 31, 2022, approximately.

## Data Availability

Any data required to support the protocol can be supplied on request.

## Abbreviations

NCV: Novel coronavirus-19 vaccine
AEFI: Adverse events following immunization
AA: Auricular acupressure
SAA: Sham auricular acupressure
WL: Wait-list
